# Zona Pellucida Genes and Proteins: Essential Players in Mammalian Oogenesis and Fertility

**DOI:** 10.3390/genes12081266

**Published:** 2021-08-19

**Authors:** Paul M. Wassarman, Eveline S. Litscher

**Affiliations:** Dept. Cell, Developmental, and Regenerative Biology Icahn School of Medicine at Mount Sinai, Annenberg Building-Box 1020, One Gustave L. Levy Place, New York, NY 10029, USA; eveline.litscher@mssm.edu

**Keywords:** zona pellucida, mammalian oogenesis, gene expression, proteins, zona pellucida domain, polymerization, fibrils, gene targeting, gene mutations, female fertility

## Abstract

All mammalian oocytes and eggs are surrounded by a relatively thick extracellular matrix (ECM), the zona pellucida (ZP), that plays vital roles during oogenesis, fertilization, and preimplantation development. Unlike ECM surrounding somatic cells, the ZP is composed of only a few glycosylated proteins, ZP1–4, that are unique to oocytes and eggs. ZP1–4 have a large region of polypeptide, the ZP domain (ZPD), consisting of two subdomains, ZP-N and ZP-C, separated by a short linker region, that plays an essential role in polymerization of nascent ZP proteins into crosslinked fibrils. Both subdomains adopt immunoglobulin (Ig)-like folds for their 3-dimensional structure. Mouse and human *ZP* genes are encoded by single-copy genes located on different chromosomes and are highly expressed in the ovary by growing oocytes during late stages of oogenesis. Genes encoding ZP proteins are conserved among mammals, and their expression is regulated by *cis*-acting sequences located close to the transcription start-site and by the same/similar *trans*-acting factors. Nascent ZP proteins are synthesized, packaged into vesicles, secreted into the extracellular space, and assembled into long, crosslinked fibrils that have a structural repeat, a ZP2-ZP3 dimer, and constitute the ZP matrix. Fibrils are oriented differently with respect to the oolemma in the inner and outer layers of the ZP. Sequence elements in the ZPD and the carboxy-terminal propeptide of ZP1–4 regulate secretion and assembly of nascent ZP proteins. The presence of both ZP2 and ZP3 is required to assemble ZP fibrils and ZP1 and ZP4 are used to crosslink the fibrils. Inactivation of mouse *ZP* genes by gene targeting has a detrimental effect on ZP formation around growing oocytes and female fertility. Gene sequence variations in human *ZP* genes due to point, missense, or frameshift mutations also have a detrimental effect on ZP formation and female fertility. The latter mutations provide additional support for the role of ZPD subdomains and other regions of ZP polypeptide in polymerization of human ZP proteins into fibrils and matrix.

## 1. Introduction

Extracellular matrix (ECM) that surrounds most animal cells can affect cellular adhesion and migration, cell-to-cell communication, as well as gene expression, differentiation, and morphogenesis [1]. ECM consists of proteoglycans (e.g., hyaluronic acid, heparin-, chondroitin-, and keratin-sulfate) and fibrous proteins (e.g., collagens, elastins, fibronectins, and laminins) [2,3,4]. On the other hand, ECM of mammalian oocytes and eggs, the zona pellucida (ZP), is composed of a unique set of glycosylated proteins, ZP1–4, that differ from proteins present in somatic cell ECM [5,6,7].

Each ZP protein has a zona pellucida domain (ZPD) that consists of ≈270 amino acids (aa), 8 or 10 conserved cysteine (Cys) residues present as intramolecular disulfides, and two subdomains, ZP-N and ZP-C. These subdomains adopt immunoglobulin (Ig)-like folds and are connected to each other by a short, protease-sensitive linker region [7,8,9,10,11]. Subdomain ZP-N is involved in polymerization of nascent ZP proteins into fibrils, as well as in polymerization of many other ZPD-containing proteins, such as tectorin, uromodulin, mesoglein, and cuticlins, into fibrils and matrices. Mutations in *ZPD* genes can result in severe human pathologies such as vascular disease, renal disease, deafness, cancer, or infertility. Although the ZP and somatic cell ECM consist of different proteins, they have certain properties in common, such as viscoelasticity that can affect cellular behavior. It has been proposed that ZP proteins self-aggregate into fibrillar structures via cross-β-sheets, similar to the structure of amyloids.

A ZP first appears as oocytes begin to grow, continues to thicken as oocytes increase in size, and is from ≈2 to ≈20 µm thick for fully grown oocytes from different mammals, e.g., the human egg ZP (hZP; ≈18 µm width) is about 3 times thicker than the mouse egg ZP (mZP; ≈6 µm width). The ZP is a viscoelastic ECM permeable to large macromolecules, (e.g., antibodies, enzymes, and small viruses) and consists of long, crosslinked fibrils that are polymers of ZP proteins. A variety of agents that do not break covalent bonds dissolve the ZP indicating that its components are held together by non-covalent interactions.

The ZP plays vital roles during oogenesis, fertilization, and preimplantation development. For example, it supports the health and growth of oocytes and follicles during oogenesis, provides species-restricted receptors for binding of free-swimming sperm to eggs during fertilization, undergoes both physical and biological changes that help to prevent polyspermy following fertilization, and protects preimplantation embryos as they traverse the female reproductive tract on their way to the uterus. In this context, it has been demonstrated that either inactivation of *mZP* genes or mutation of *hZP* genes can have a deleterious effect on ZP formation during oogenesis and can result in female infertility.

## 2. Mammalian Oogenesis

The ovary was recognized as an anatomical entity by Herophilus in ~300 BC and was described in some detail by Soranus in ~50 AD [12]. Regnier de Graaf (1641–1673) recognized in ~1670 that eggs came from the ovary, but concluded incorrectly that the entire follicle, egg plus surrounding follicle cells, was an egg. This was rectified by William Cruickshank (1745–1800) in ~1795, however, it remained for Karl Ernst von Baer (1792–1876) in ~1827 to elucidate the relationship between the egg and the remainder of the ovarian follicle. von Baer was the first to use the term ZP when describing human eggs in 1827 [13] and by the 1840s the term ZP had gained widespread use among embryologists.

### 2.1. Meiosis during Oogenesis

**Figure 1:**
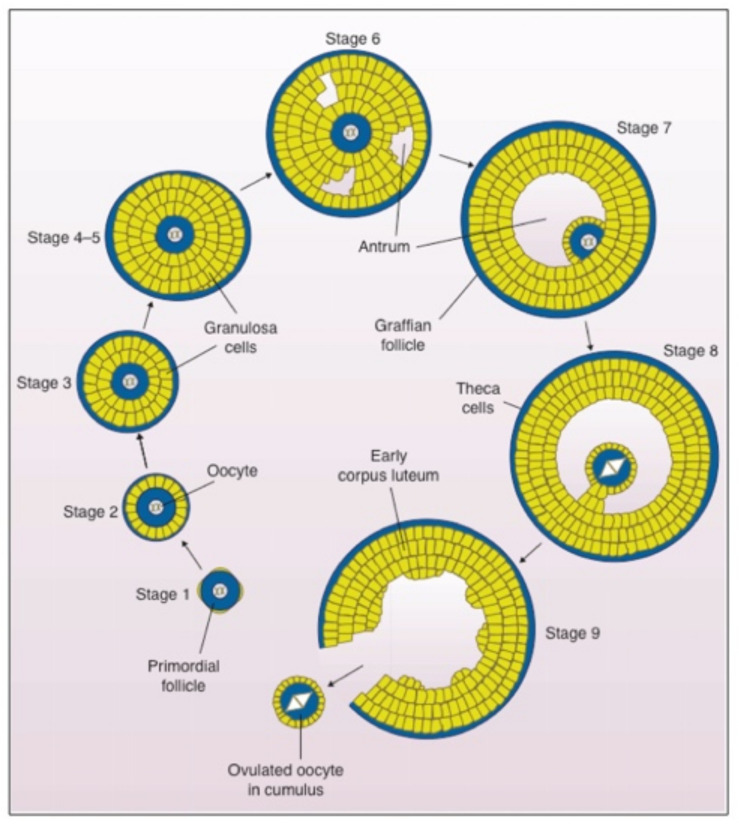
Stages of follicular growth in mammals. Follicular growth begins with stage-1 primordial follicles in the ovary, which consists of a non-growing oocyte (blue) surrounded by a few epithelial-like somatic cells (yellow). As growth is initiated in stage-2 follicles, the somatic cells or granulosa cells (yellow) become cuboidal. During stages-3–5, the granulosa cells proliferate while the oocyte continues to increase in diameter and lays down a thin ZP (blue; outermost layer) that continues to thicken throughout oocyte growth. In stage-6, a fluid-filled cavity or antrum begins to form and by stage-8 the antrum is complete. Surrounded by a relatively thick ZP, the fully grown oocyte sits at the end of a stalk of granulosa cells and is surrounded by several layers of cumulus cells (yellow; stage-8). At stage-9, the fully grown oocyte (blue), which has arrested at metaphase II of meiosis, is ovulated into the oviduct surrounded by cumulus cells (yellow). The follicle that is left behind becomes an endocrine gland, the corpus luteum, that supports pregnancy. In female mice it takes ≈2–3 weeks for this developmental process to be completed. This figure is adapted from [14], Figures 2–9, with permission from Cambridge University Press, CSIRO 2002.

**Figure 2:**
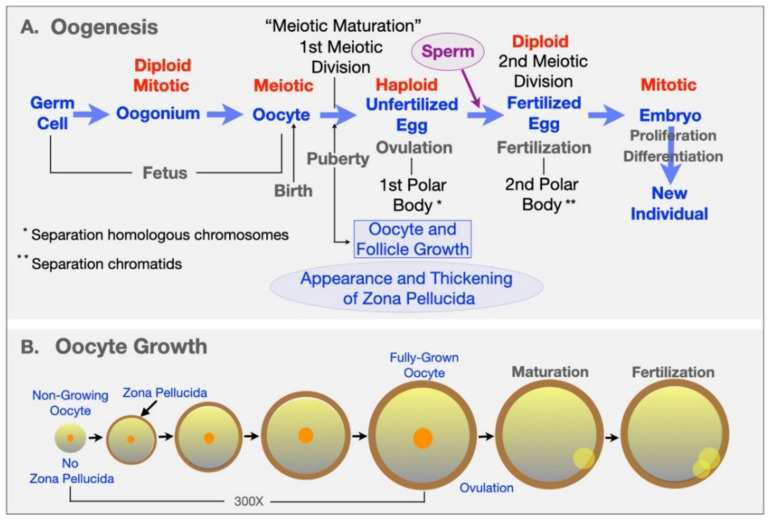
(**A**) Schematic representation of steps involved in the conversion of female germ cells in the mouse fetus to fertilized eggs in the adult mouse. In the fetus primordial germ cells convert to mitotic oogonia and then to meiotic oocytes with crossing over and recombination. Soon after birth, all oocytes are arrested in meiosis at diplotene (dictyate) of the first meiotic prophase. At puberty, during each reproductive cycle, some mouse oocytes grow ≈ 300-fold in volume over ≈2–3 weeks, their surrounding follicle cells proliferate and differentiate, and fully grown oocytes surrounded by cumulus cells are ovulated. At about the time of ovulation, oocytes undergo meiotic maturation with emission of a first polar body following separation of homologous chromosomes (1st meiotic division). In this manner fully grown oocytes become haploid unfertilized eggs. Upon fusion with a single sperm, fertilized eggs emit a second polar body following separation of chromatids (2nd meiotic division) but are restored to a diploid state by the haploid sperm genome. (**B**) Schematic representation of ZP production during oocyte growth in mice. Non-growing oocytes lack a ZP, but as soon as oocyte growth begins, they lay down a ZP that continues to thicken throughout the growth phase (≈2–3 weeks; ≈300-fold increase in oocyte volume) and results in a 6.2 ± 1.9 μm thick ZP around fully-grown oocytes and ovulated eggs. The ZP remains around the early embryo until the expanded blastocyst stage when the embryo hatches from the ZP and implants in the uterus.

Oogenesis is the process by which unfertilized eggs are produced in the ovary (Figure 1 and Figure 2). It begins early in fetal development with formation of primordial germ cells (PGCs) that are converted into oogonia (mitotic) and then into oocytes (meiotic) in the fetus and finally into unfertilized eggs in sexually mature adults [14,15,16] (Figure 2A). Following gonadal sex differentiation, embryonic day 12–13 in mice, conversion of PGCs into oogonia is completed. Oogonia enter first meiotic prophase and are converted into oocytes at various stages of meiotic prophase. In mice it takes about 4 days for oocytes to complete nuclear progression from leptotene to diplotene of meiosis with chromosomes exhibiting chiasmata due to recombination and crossing over. Following birth, most oocytes are arrested in late diplotene, the dictyate stage, where they remain until stimulated to resume meiosis at the time of ovulation. This pool of small, non-growing oocytes that lack a ZP is the sole source of unfertilized eggs in the sexually mature adult.

### 2.2. Oocyte and Follicle Growth

Mouse oocytes undergo a ≈300-fold increase in volume, from ≈0.9 (≈12 µm diameter) to ≈270 pl (≈80 µm diameter), during their 2–3-week growth phase [17] (Figure 2B). Commencement of oocyte growth is regulated within the ovary, with the number of oocytes entering their growth phase being a function of the size of the pool of non-growing oocytes. Non-growing oocytes are enclosed within several squamous follicular cells that will undergo extensive mitotic proliferation during oocyte growth and give rise to a large Graafian follicle from which an unfertilized egg is ovulated. Most follicle growth occurs after the oocyte has ceased growing and gives rise to granulosa cells, thecal cells, and a large follicular cavity or antrum (Figure 2A). The mouse Graafian follicle, with the oocyte in an acentric position surrounded by layers of granulosa or cumulus cells, is ≈600 µm in diameter and consists of ≈50,000 cells. The human Graafian follicle is 20–25 mm in diameter, consists of ≈50,000,000 cells, and contains an oocyte ≈120 µm in diameter.

### 2.3. Meiotic Maturation of Oocytes

In sexually mature animals, fully grown oocytes in Graafian follicles are hormonally stimulated to resume meiosis and complete the first meiotic reductive division just prior to ovulation (Figure 2A). Oocytes undergo nuclear progression from dictyate to metaphase II (meiotic maturation) and remain at this stage in the oviducts until stimulated to complete meiosis by sperm–egg fusion (fertilization). Meiotic maturation involves dissolution of the oocyte’s nucleus or germinal vesicle (GV), condensation of chromatin into distinct bivalents, separation of homologous chromosomes and emission of a first polar body, and arrest of meiosis with chromosomes aligned on the metaphase II spindle [18]. Eggs complete meiosis, with separation of chromatids and emission of a second polar body, upon fertilization and are restored to a diploid state by fusion with a single sperm.

### 2.4. Ultrastructural Changes during Oocyte Growth

Throughout reproductive life ovaries contain non-growing oocytes arrested in dictyate of the first meiotic prophase. Only fully grown oocytes resume meiosis and are ovulated during each estrous cycle, with follicular growth controlled by pituitary gonadotropins, luteinizing hormone (LH), and follicle-stimulating hormone (FSH). Oocytes undergo tremendous enlargement during growth, a period of intense metabolic activity as reflected in marked changes in oocyte ultrastructure [19]. For example: (i) The diameter of the GV increases several fold, as does the ratio of cytoplasmic to nucleoplasmic volume. (ii) The diameter of the nucleolus increases several-fold accompanied by changes in its fine structure due to intense ribosomal-RNA synthesis. (iii) The number of mitochondria increases significantly, and mitochondria undergo a major shape change from elongated to round or oval. (iv) The ultrastructure of the Golgi changes dramatically with increased numbers of swollen, stacked lamellae due to increased processing of secretory products, such as cortical granule components and ZP proteins. (v) The ZP appears first as localized pockets of fibrils around growing oocytes, but soon the fibrils coalesce to form a uniform, thickening ECM. (vi) Long processes from the innermost follicle cells surrounding oocytes (corona radiata) penetrate through the ZP and form gap junctions with oocyte microvilli. These junctions (pore size ≈ 15 Å) allow passage of small molecules (<1000 MW), such as amino acids, nucleotides, and metabolites, into oocytes from the surrounding syncytium of granulosa cells [20]. Such communication between growing oocytes and granulosa cells is necessary for normal follicular development (see Section 7.1).

## 3. *ZP* Gene Expression

### 3.1. Transcription of ZP Genes during Oocyte Growth

Expression of mammalian *ZP* genes encoding ZP1–4 is both female- and organ-specific [21,22,23,24]. mZP and hZP transcripts have unusually short 5′- and 3′-untranslated regions [22,25,26]. mRNA encoding mZP proteins is undetectable (<1000 copies/oocyte) in non-growing oocytes, but increases to hundreds-of-thousands of copies per oocyte in mid-stage growing (≈30–70 µm diameter) and fully grown oocytes (≈80 µm diameter) [27]. It has been estimated that in mid-stage growing oocytes (50–60 µm diameter) mZP transcripts represent≈1.5% of the total polyA^+^-RNA (≈1000 fg ZP2 and ≈400 fg ZP3 transcripts) [22,27,28]. Fully grown mouse oocytes contain ≈195 ± 20 fg of ZP3 mRNA/oocyte (≈2.4 × 10^5^ copies of ZP3 mRNA/oocyte) which represents ≈0.27% of the oocyte’s poly(A)^+^RNA. mZP mRNA levels fall to almost undetectable levels (≈1% of peak levels during oocyte growth) following meiotic maturation and ovulation of unfertilized eggs and remain so following fertilization. The molar ratio of ZP2 and ZP3 transcripts is about 2:1 throughout oocyte growth [22,25,28]. At ovulation transcription is terminated in oocytes, mZP transcripts are shortened by de-adenylation of their poly(A) tails, and about one-half of total mRNA (i.e., poly(A)^+^RNA) present in fully grown oocytes is degraded [21,23,26]. mZP3 mRNA is selectively degraded during ovulation since>95% of mZP3 mRNA present in fully grown oocytes is lost; ovulation is a period when transcription by oocytes is terminated.

### 3.2. Location and Size of mZP and hZP Genes

mZP and hZP are encoded by single-copy genes located on different chromosomes and their expression is coordinately regulated [22,25,29,30,31,32,33,34]. *mZP1*, *mZP2*, and *mZP3* genes are located on chromosomes 19 (7.36 cM), 7 (11.3 cM), and 5 (9.2 cM), respectively, vary in length from 6.5 (ZP1) to 18.5 (ZP2) kb, and contain 12 (ZP1), 18 (ZP2), and 8 (ZP3) exons. *mZP4* is a pseudogene located on chromosome 13; pseudogenization of *mZP4* is restricted to the subgenus *Mus* [35]. *hZP1*, *hZP2*, *hZP3*, and *hZP4* genes are located on chromosomes 11 (11q12.2), 16 (16p12.3-p12.2), 7 (7q11.23), and 1 (1q43), respectively, vary in length from 8.1 (ZP1) to 18.3 (ZP3) kb, and contain 12 (hZP1), 19 (hZP2), 8 (hZP3), and 12 (hZP4) exons. Genomic organization of ZP loci is identical in oocytes and somatic cells, however, ZP genes are hypomethylated in oocytes where they are expressed as compared to somatic cells where they are not expressed [36].

### 3.3. Conservation of ZP Genes

Genes encoding ZP proteins are conserved among mammals with distinct domains defined by exon/intron boundaries that contain consensus splice donor/acceptor sites. It is likely that expression of *ZP* genes in all mammals is regulated by similar *cis*-acting sequences and *trans*-acting factors. For example, there is a high degree of conservation between coding regions and between the first 300 bp of the 5′-flanking-regions of *mZP* and *hZP* genes [22,31,32,33,34,36,37,38]. Conservation of sequences between the first 300 bp of the mZP and hZP gene promoters enable the hZP promoter to utilize the transcriptional machinery of mouse oocytes [39]. Mutation of a 12 bp sequence element (element 4) located upstream of the transcription start site of *mZP3*, *hZP2*, or *hZP3* reduces reporter gene activity to ≈1–4% of wild type activity [40]. 

### 3.4. Cis-Acting Sequences Regulate Transcription

*Cis*-acting sequence elements in the 5’-flanking-region of *ZP* genes regulate oocyte-specific expression [22,23,24,41]. Transgenes containing different amounts of *mZP3* 5′-flanking-region, from 470 to 6500 nt, fused to the coding region of the firefly luciferase gene, target expression of luciferase solely to growing oocytes. On average, ovaries excised from 15-day-old transgenic females containing 6500 nt of *mZP3* 5′-flanking-region, exhibit ≈ 6000 times more luciferase activity (3.98 × 10^5^ luciferase units; 175 pg luciferase) than other tissues (62 luciferase units). Intervening *mZP3* sequences absent from these transgenes improve transcriptional efficiency by 10- to 100-fold. Comparisons of levels of expression of different *mZP3-luciferase* transgenes suggest that sequences between −153 and −470 nt in the *mZP3* 5′-flanking-region greatly affect the level of expression of luciferase.

### 3.5. Trans-Acting Factors Regulate Transcription

*mZP* and *hZP* genes have a potential TATA-box just upstream of the transcription start-site (e.g., at-31bp in *mZP2* and -21bp in *mZP3*) and *cis*-acting sequence elements have potential binding sites for several *trans*-acting factors [22]. For example, the sequence CANNTG, an E box domain located ≈200 bp upstream of the transcription start site of both *mZP* and *hZP* genes, is identical to the consensus sequence required for binding of proteins that belong to the basic helix-loop-helix (bHLH) family of DNA binding-proteins [42,43]. This site is involved in oocyte-specific expression of *ZP* genes when *ZP* gene activating protein-1 (ZAP-1), possibly a complex of proteins, binds to DNA [44]. The abundance of ZAP-1 during oogenesis mirrors the pattern of mZP gene expression. Similarly, an oocyte specific protein-1 (OSP-1), binds to a minimal sequence TGATAA located within the first 100 bp (−97 to −93 nt) of the *mZP3* gene promoter [45]. Like ZAP-1, the abundance of OSP-1 during oogenesis mirrors the pattern of *mZP* gene expression. The minimal sequence recognized by OSP-1 is identical to the consensus sequence A/TGATAG/A that is the binding motif for the GATA multigene family of DNA binding-proteins [46].

## 4. ZP Protein Characteristics

### 4.1. mZP and hZP Proteins

**Figure 3:**
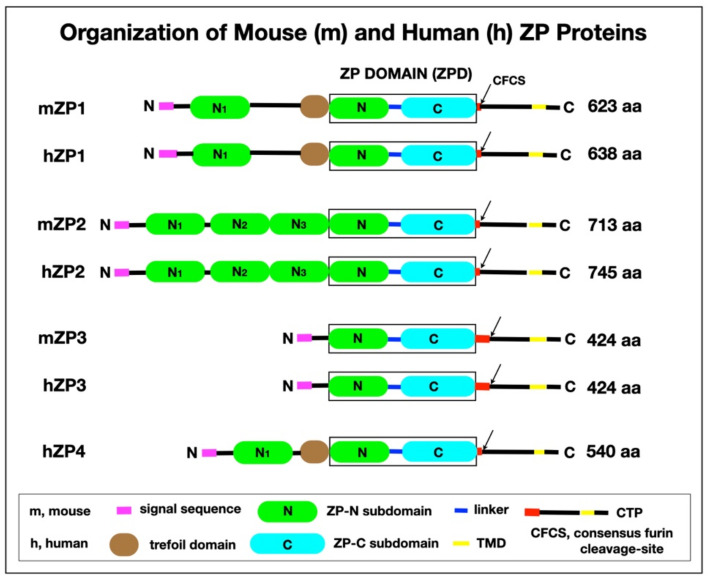
Organization of mZP and hZP proteins. The polypeptide has a signal sequence (magenta), a ZPD that consists of ZP-N (green) and ZP-C (cyan) subdomains, and a linker region (blue); a CFCS (arrow); a TMD) (yellow); and a short CT (black) in the CTP. mZP1, hZP1, and hZP4 also have a TD (brown). mZP2 (713 aa) has 3 additional ZP-N subdomains, N1–N3 (green), between the N-terminus of the polypeptide and the ZPD. mZP1 (623 aa), hZP1 (638), and hZP4 (540 aa) have one additional ZP-N subdomain, N1 (green), between the N-terminus of the polypeptide and the TD. mZP3, the smallest of the 3 mouse ZP proteins (424 aa), consists primarily of a ZPD. mZP1 and mZP2 have only 3 or 4 aa between the ZPD and the CFCS (red), whereas mZP3 has 47 aa, which is a region of positive Darwinian selection during evolution (red) [63] and is the binding site for acrosome-intact, free-swimming sperm during fertilization [64].

The ZP of all mammalian eggs, from monotremes to eutherians, consists of 3–4 glycosylated proteins (Figure 3). mZP (*Mus musculus*) is composed of three proteins, mZP1-3 [5,6,7,47,48], and hZP has an additional protein, hZP4 [49,50,51,52]. mZP1-3 have apparent MWs of approximately 200, 120, and 83 kDa, respectively, and hZP1-4 have MWs of approximately 200, 120, 58, and 65 kDa, respectively (based on non-reducing, SDS-gel electrophoresis). Comparison of human and chimpanzee ZP1-4 aa sequences suggests they share ≈99% average identity, whereas human and rodents share ≈65% average identity [10,53,54,55]. Generally, the primary structures of ZP2- and ZP3-related proteins from different mammals are well conserved having ≈65–98% average identity, whereas ZP1-related proteins are conserved to a lesser degree having ≈40% average identity. It should be noted that an alternative nomenclature for ZP proteins occasionally has been used in which ZP1, ZP2, and ZP3 are referred to as ZPB, ZPA, and ZPC, respectively.

In mice and humans, ZP1 is a dimer of two identical polypeptides linked by a single intermolecular disulfide, whereas ZP2 and ZP3 are monomers [5,6,8,22,53,55]. ZP2 and ZP3 are present in roughly equimolar amounts in the ZP, whereas ZP1 and ZP4 are the least abundant proteins. ZP1 and ZP4 are homologs, but ZP4 is a monomer and lacks an intermolecular disulfide. mZP1 and hZP1 have a proline (Pro)-rich N-terminal region (mZP1 21% Pro; hZP1 17% Pro), whereas hZP4 (2% Pro) does not [7]. This Pro-rich region may provide the flexibility that contributes to the elasticity of the ZP prior to fertilization. ZP4 is shorter than ZP1 since it lacks the Pro-rich, N-terminal region of ZP1, but otherwise is comparable with respect to domain organization. Unlike ZP2 and ZP3, ZP1 and ZP4 have a trefoil domain (TD), a 3-loop compact structure with three intramolecular disulfides [56]. All ZP proteins are glycosylated to different extents with asparagine-(N-) and serine/threonine-(O-) linked oligosaccharides. mZP1-3 possess 4, 6, and 5 N-linked oligosaccharides, respectively [5,52,57,58,59,60,61,62]. While ZP polypeptides have neutral isoelectric points (pI 6.5–6.7), their oligosaccharides may be sialylated and sulfated such that ZP proteins tend to be acidic (pI 4.1–5.2) and migrate as broad bands during electrophoresis on denaturing gels.

### 4.2. ZPD of ZP Proteins

A relatively large structural element, the ZPD, was identified in ZP polypeptides nearly 30 years ago [65] (Figure 3). The ZPD arose more than 600 million years ago and has been found in many proteins that have diverse functions, from receptors to mechanical transducers, in a wide variety of multicellular organisms, from jelly fish to humans [7,8,9,10,11]. Mouse ZP proteins are prototypical ZPD proteins with a ZPD located at aa 271–542 for mZP1, aa 364–630 for mZP2, and aa 45–304 for mZP3. The ZPD consists of ≈270 aa in the polypeptide’s C-terminal region and has 8 or 10 conserved Cys residues present in four intramolecular disulfides. It has two subdomains, ZP-N (≈100 aa) and ZP-C (≈145 aa), separated by a short linker-region (≈27 aa) (Table 1). For different ZPD proteins the linker-region between ZP-N and ZP-C can be either flexible, as found in ZP2 and ZP3, or rigid, as found in uromodulin [66]. It has been postulated that differences in plasticity of linker-regions modulate polymerization of structurally similar ZPD proteins into either homo- or hetero-polymers.

Protein domains like the ZPD are evolutionary units that can be duplicated and recombined. Pairs of domains are usually found in one sequential order (A → B or B → A), but never in both. This is the case with the ZPD as ZP-N and ZP-C are always present in one order, ZP-N → ZP-C, and not in the other, ZP-C → ZP-N, although ZP-N can be found by itself in some proteins [8,10]. In this context, it has been proposed that the two ZPD subdomains should be designated as independent domains [67]. ZP1 and ZP4 have one extra ZP-N subdomain (N1) and ZP2 has three extra ZP-N subdomains (N1, N2, and N3) at the N-terminus of their polypeptides [68]. ZP-N is used for polymerization of nascent ZP proteins [69,70] and other extracellular ZPD proteins, such as tectorin [71] and uromodulin [72], consistent with an earlier suggestion that the ZPD plays a role in protein polymerization [73,74]. It is likely that ZPD proteins are derived from a common ancestral gene. It has been proposed that a first duplication event in evolution gave rise to ZP3 and an ancestral ZP gene subsequently duplicated several times and evolved into all other ZP genes [55,63,75,76,77].

### 4.3. Proteolytic Processing of ZP Proteins

Nascent ZP proteins have an N-terminal signal sequence (SS; ≈20–30 aa) that targets them to the secretory pathway, a ZPD (≈270 aa) with a short internal hydrophobic patch (IHP), and a C-terminal propeptide (CTP) required for secretion of ZP proteins [7,8,78,79,80] (Figure 3, Table 2). The CTP has a consensus furin cleavage-site (CFCS; Arg-X-X-Arg or Arg-X-Arg/Lys-Arg), a short, external hydrophobic patch (EHP), a hydrophobic transmembrane domain (TMD; ≈20 aa), and a short cytoplasmic tail (CT). ZP1 and ZP4 also have a TD. Polypeptides of nascent ZP proteins are processed in oocytes by proteolytic removal of the N-terminal SS as they move from endoplasmic reticulum to Golgi and by proteolytic removal of the CTP at the plasma membrane. Unprocessed polypeptides of mZP1-3 are 623, 713, and 424 aa in length and processed polypeptides are 526, 599, and 329 aa in length, respectively. Unprocessed polypeptides of hZP1-4 are 638, 745, 424, and 540 aa in length and processed polypeptides are 528, 602, 328, and 445 aa in length, respectively.

### 4.4. Secretion of ZP Proteins

Nascent ZP proteins are localized to large secretory vesicles (SV) ≈2.3 µm diameter, or ≈10 times larger than SV of somatic cells (≈0.1–0.2 µm diameter) [81,82]. Doughnut-shaped SV with an empty lumen in the middle originate from the Golgi and contain nascent ZP proteins. ZP proteins insert their TMD into SV-membrane prior to fusion of SV with the oocyte’s plasma membrane. The ZP thickens from the inside to the outside since ZP proteins are deposited into the innermost layer of the growing matrix [81]. In mice, the ZP of fully-grown mouse oocytes varies considerably in thickness, from ≈4.3 to ≈8.1 μm, suggesting that the assembly of the ZP matrix is a stochastic process. Apparently, the thickness of the ZP does not affect the ability of free-swimming sperm to bind to unfertilized eggs [83] (see Section 7.2). Note that not all nascent ZP proteins are incorporated into the ZP, with ≈50% passing through the ZP unassembled under certain conditions [84].

## 5. ZP Protein 3-Dimensional Structure

The 3-dimensional structures of several ZPD proteins have been determined by X-ray diffraction [66,85,86,87,88,89,90,91]. These include the ZP-N subdomain of mouse ZP3 (2.3 Å) [85], full-length chicken ZP3 (2.0 Å) [86], ZP-C subdomain of mouse ZP2 (2.25 Å) [66,89], and homodimers of chicken ZP1 (2.7 Å resolution) [91]. Electron cryo-microscopy has also been used to solve the structure of fibrils of uromodulin, another ZPD protein [92].

### 5.1. ZPD 3-Dimensional Structure

**Figure 4:**
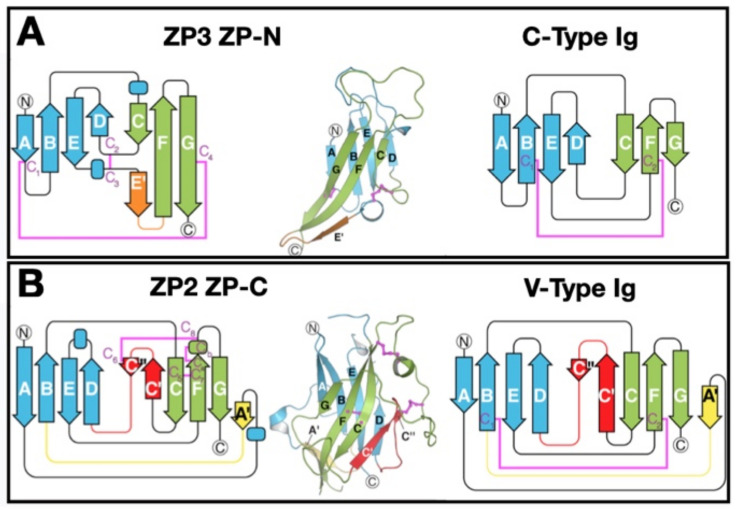
Three-dimensional structures of ZPD subdomains ZP-N and ZP-C that are related to C- and V-type Ig-like domains. (**A**) ZP3 subdomain ZP-N and C-type Ig-like domains. β-strands are labeled using Ig terminology; helices are indicated by rectangles. Opposing β-sheets 1 and 2 are blue and green, respectively, with termini circled. The E′ strand is orange and disulfides magenta. (**B**) ZP2 ZP-C and V-type Ig-like domains. As in panel A, except for the additional A′ and C′/C″ strands that are yellow and red, respectively. This figure was adapted with permission from L. Jovine ([11], Figure 4), Copyright 2018.

Both the ZP-N and ZP-C subdomains adopt immunoglobulin (Ig)-like folds despite the complete absence of sequence identity [11,66,85,89,90,91,92,93] (Figure 4). The ancestral gene for the Ig superfamily may have originated ≈ 750 million years ago in invertebrates as a primitive sandwich-like fold used in extracellular recognition systems (cell–cell and/or cell–matrix) [94]. The ZP-N and ZP-C sandwich-like folds resemble C- and V-type Ig-like domains, respectively [11]. The ZP-N fold consists of an antiparallel sandwich of two β-sheets made up of eight strands of polypeptide that enclose a hydrophobic core, with two buried disulfides that clamp both sides of the sandwich. The ZP-C fold also consists of a β-sandwich of stacked β-sheets, one with four and the other with six strands, that resemble a Greek key-like motif characteristic of Ig-like domains [95]. The similar structures of ZP-N and ZP-C suggests that the ZPD may have arisen by duplication of an ancestral gene encoding a protein with one ZP-N subdomain.

### 5.2. ZP Protein Arrangement

Structural data has revealed that 2 ZP3 molecules are present as homodimers in an antiparallel orientation, forming an asymmetric structure [11]. ZPDs of ZP3 molecules interact electrostatically between ZP-N and ZP-C of opposing proteins, ZP-N(1):ZP-C(2) and ZP-N(2):ZP-C(1), with no ZP-N(1):ZP-N(2) or ZP-C(1):ZP-C(2) present. In uromodulin, ZP-N and ZP-C are arranged in a similar fashion to the ZP3-fold, with the same disulfide connections and a conserved tyrosine residue in the ZP-N. In tectorin, a ZPD protein present in the tectorial membrane of the ear, mutation of this tyrosine results in hearing loss [96].

As described above, the ZP-N subdomain adopts an Ig-like β-sandwich fold with each β-sheet made up of four β-strands connected by two disulfides (e.g., mZP3, C46 → C139 and C78 → C98) [11,93]. Between the A and G β-strands is an exposed hydrophobic surface area that could enhance successive monomer interactions to generate polymers. However, other contact sites, such as those between β-strands of IHP segments and between E′ β-strands of adjacent ZP-N subdomains, have been noted. This suggests that ZP-N subdomains of adjacent ZP proteins, such as in ZP2-ZP3 dimers, interact and cause polymerization of ZP fibrils.

### 5.3. ZP Proteins as Functional Amyloids

Amyloid fibrils consist of a stack of β-strands that generate a β-sheet, with protofilaments consisting of 2 β-sheets [97]. Examination of peptide analogs of human and fish ZP proteins has revealed that ZP fibrils possess amyloidogenic properties with short peptide sequences that stack atop one another as β-strands to form a β-sheet.

The analogy between amyloids and ZP fibrils comes from biophysical descriptions of the fibrils and an hZP1 structural model based on the ZP-N subdomain predicts how this subdomain could assemble into fibrils [98]. Analyses with peptide analogs of hZP2-4 that have ZP-N subdomains with the same 3-dimensional structure, yielded results similar to those with hZP1 [99]. Furthermore, mZP1-3 fibril-forming sequences were identified, many of which were located in β-strands in the ZP-N subdomain [100]. These and other results suggest that ZP1-4 are amyloidogenic proteins and the ZP is a functional amyloid [101].

## 6. ZP Fibril Assembly and Arrangement

### 6.1. Sequence Elements Regulate ZP Protein Polymerization

**Figure 5:**
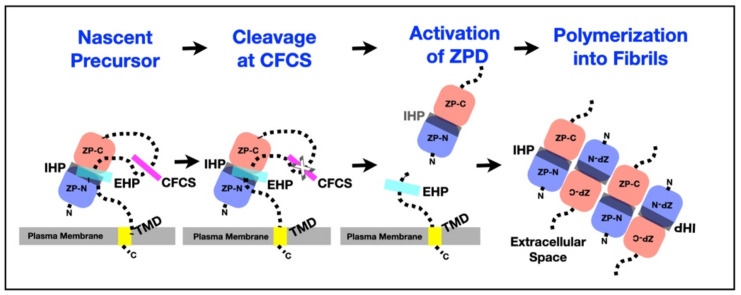
A general mechanism for assembly of nascent ZP proteins. In all ZPD precursor proteins, the ZPD consists of 2 subdomains, ZP-N (blue) and ZP-C (pink). The subdomains are followed by a CTP that contains a CFCS (magenta), an EHP (cyan), and a TMD (yellow). Precursors do not polymerize within the cell, either as a result of direct interaction between the EHP and IHP (gray) or because they adopt a conformation dependent on the presence of both hydrophobic patches. Proteolytic processing at the CFCS (marked by a cross) leads to dissociation of mature proteins from the EHP and activation of the ZPD for polymerization into fibrils and matrix.

Secretion of ZP proteins is dependent of sequence elements located in the CTP, between the CFCS and TMD, and in the ZPD (Figure 5, Table 2). Nascent ZP proteins have two hydrophobic patches, an EHP in the CTP and an IHP in the ZPD [7,11,78,79,80,81,102]. The EHP and IHP interact and lock nascent ZP proteins in a conformation that prevents formation of ZP fibrils in growing oocytes prior to excision of the CTP at its CFCS. Loss of the EHP results in an unlocking of the ZPD and enables polymerization of ZP proteins to take place [78,79,80,81]. Secretion of nascent ZP proteins by growing oocytes is inhibited when either the EHP or IHP is mutated in the absence of a TMD [78,79,80,81]. The IHP, CFCS must be cleaved for normal levels of secretion of ZP proteins and nascent mZP proteins accumulate in the endoplasmic reticulum when cleavage fails to occur [103,104,105]. The EHP, IHP, CFCS, and TMD regulate incorporation of nascent ZP proteins into the ZP by a coupling of proteolysis and polymerization. The TMD can be replaced by an unrelated TMD without altering its function [80]. Cleavage of inhibitory sequences from protein precursors with concomitant exposure of polymerization elements regulates polymerization of several other kinds of proteins, such as fibrinogen [106], fibrillin [107], and tau protein [108].

### 6.2. ZP Fibril Arrangement in Layers

**Figure 6:**
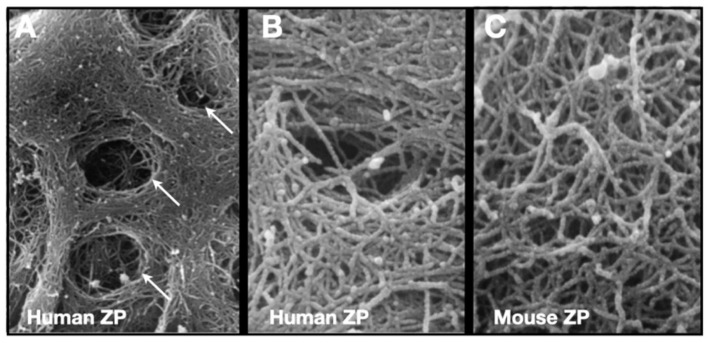
Scanning electron micrographs of the surface of human and mouse oocytes. (**A**) Human oocyte showing the presence of many pores (9000× magnification) on the outer surface of the ZP. (**B**) Higher magnification of a human oocyte showing the fibrillar organization of the ZP (50,000× magnification); fibrils are 0.1–0.4 μm long and 10–14 nm wide. (**C**) Outer surface of a mouse oocyte showing the fibrillar organization of the ZP (50,000× magnification). Samples were treated with saponin–ruthenium red–osmium–thiocarbohydrazide to reveal ZP fibrils. This figure was adapted with permission from G. Familiari ([113], Figure 3), Copyright 2012.

The mZP consists of crosslinked fibrils ≈7–8 nm in width and several micrometers in length and have a ZP2-ZP3 dimer located every ≈14–15 nm along the fibrils [64,109,110,111]. The surface of the ZP is covered with many pores (≈50 pores/ZP), giving it a laminated, spongelike appearance “somewhat reminiscent of layers of sliced Swiss cheese put down irregularly on top of one another” [112,113] (Figure 6). mZP and hZP are multilayered structures with fibrils in the inner and outer layers oriented perpendicular and parallel, respectively, to the oolemma, and fibrils in the intervening layer oriented randomly [114,115,116]. Inner layer fibrils are more densely packed than those in the outer layer, and this is consistent with the finding that nascent ZP proteins are deposited into the innermost layer of the ZP [81]; consequently, nascent matrix deposited around small oocytes will end up close to the surface of the ZP of fully-grown oocytes. Matrix near the ZP surface must undergo considerable stretching in order to accommodate the several fold increase circumference during oocyte growth. For example, the circumference of mouse oocytes undergoes a 6- to 7-fold increase during oocyte growth and stretching matrix closest to the ZP surface may lead to reorientation of fibrils, from perpendicular to parallel with respect to the oolemma.

## 7. *mZP* Genes and Female Fertility

Results of experiments in which antisense oligonucleotides directed against either mZP2 or mZP3 mRNAs were injected into growing mouse oocytes strongly suggest that mZP2 and mZP3 are dependent upon each other for incorporation into the ZP [117]. To extend these observations, gene targeting was used to establish mouse lines in which *mZP* genes were inactivated by either homologous recombination or insertional mutagenesis and the fertility of the mice was assessed (Table 3).

### 7.1. mZP2 and mZP3 Homozygous Nulls Are Infertile

Male mice that are homozygous nulls for *mZP1*, *mZP2*, or *mZP3* are as fertile as wild type males. On the other hand, female mice that are homozygous nulls for either *mZP2* (*mZP2^-/-^*) or *mZP3* (*mZP3^-/-^*) produce eggs that lack a ZP, and these females are completely infertile [119,120,121] (Table 3). Infertility is due to a scarcity of both growing oocytes and ovulated oviductal eggs in homozygous null mice. This suggests that the presence of both mZP2 and mZP3 is absolutely required for assembly of a ZP around growing oocytes and is consistent with results of antisense experiments mentioned above [117]. The paucity of growing oocytes and follicles in ovaries of *mZP3^-/-^* mice is reflected in weight differences of ovaries from 20-day-old wild type females, 1.0 ± 0.17 mg/ovary, and ovaries from *mZP3^-/-^* females the same age, 0.26 ± 0.1mg/ovary; a 4-fold difference due to retarded oocyte growth and follicle development [122,123]. The relatively small number of growing oocytes in ovaries from homozygous null mutant mice is not intimately associated with surrounding follicle cells and ovaries contain few, if any, Graafian follicles.

It has been shown that gap junctions are present between oocytes and surrounding follicle cells at sites where follicle cell processes traverse the ZP and contact the oolemma [124,125,126]. In the absence of a ZP around oocytes from *mZP2^-/-^* and *mZP3^-/-^* mice it is likely that formation of gap junctions is severely reduced, thereby compromising the electrical and metabolic coupling between oocytes and follicle cells that is necessary for oocyte growth, follicle development, and fertility [127,128,129]. The latter is consistent with the phenotype of female mice that are homozygous nulls for gap junction proteins, such as connexin-37 and -43; these mice are infertile, and their ovaries are deficient in growing oocytes and multi-layered follicles and [130,131]. Furthermore, note the finding that as oocytes grow follicle cells elaborate enormous numbers of new transzonal projections (filopodia) that contact the oocyte surface and increase oocyte–follicle cell communication [132]. Overall, these observations with *mZP2* and *mZP3* homozygous null females suggest that the ZP may serve as a kind of glue with which to stabilize gap junctions and other contacts between oocytes and innermost follicle cells.

### 7.2. mZP3 Heterozygous Nulls Are Fertile

Female mice that are heterozygous nulls for *mZP3* (*mZP3^+/-^*) are as fertile as wild-type females, but their eggs have a thin ZP (ave. width ≈ 2.7 ± 1.2 μm) compared to the ZP of eggs from wild type females (ave. width ≈ 6.2 ± 1.9 μm) [83] (Table 3). The thin ZP contains about one-half the amount of mZP2 and mZP3 found in ZP of eggs from wild type mice. These observations suggest that the width of the ZP is not a critical parameter for either binding of free-swimming sperm to the ZP or fertilization of eggs.

### 7.3. mZP1 Homozygous Nulls Exhibit Reduced Fertility

Female mice that are homozygous nulls for *mZP1* (*mZP1^-/-^*) are fertile, but exhibit reduced fertility compared to wild type mice due to early loss of preimplantation embryos in oviducts [118] (Table 3). This loss is attributable to a ZP that is not crosslinked and, consequently, extremely fragile as cleavage-stage embryos traverse the reproductive tract on their way to the uterus. The presence of mZP2 and mZP3 in growing oocytes of *mZP1^-/-^* mice supports formation of heterodimers that can assemble into long fibrils. However, in the absence of mZP1 the fibrils are not crosslinked, creating an unusually porous ZP matrix that even permits follicle cells to enter the perivitelline space between the ZP and plasma membrane. New insights into the structural basis of hZP1/hZP4 crosslinking of the human ZP have recently been reported [91].

## 8. *hZP* Genes and Female Fertility

Female infertility has increased dramatically over the past 25 years and today it is estimated that ≈10% of married women worldwide are infertile. Nearly 65% of human infertility cases can be attributed to either male or female factors and ≈50% of infertility cases have a genetic component. In this context, some early evidence suggested that there might be a causal relationship between gene sequence variations (GSV) in *hZP* genes and female fertility [133,134,135]. For example, it was found that there was ≈1.5 times more GSV in *hZP1* and *hZP3* of women who were unsuccessful in in vitro fertilization (IVF) trials compared to women with proven fertility [133]. This finding has now been extended by a large number of case studies carried out to assess whether GSV in *hZP1-4* have an effect on female fertility. Results of these studies with human IVF patients are summarized below and in Table 4.

### 8.1. Infertile Women and Mutant hZP1 Genes

One study revealed a homozygous frameshift deletion of 8 bp in *hZP1* of women who were infertile and whose eggs lacked a ZP [143]. The deletion was predicted to result in a premature stop codon (SC) in *hZP1* and synthesis of a truncated form of hZP1; Ile390*fs*404X, a 404 aa polypeptide for mutant hZP1 versus a 638 aa polypeptide for wild type hZP1. Truncated hZP1 had the N-terminal SS, TD, and first half of the ZPD, but was missing the CTP essential for protein secretion [78,81,102,103]. As oocytes from *mZP1* homozygous null mice have a ZP, albeit a very loose and porous ZP, it was surprising that oocytes from these women lacked a ZP. However, subsequently it was reported that accumulation of truncated hZP1 in the oocyte’s cytoplasm apparently interfered with secretion of nascent hZP3 and hZP4 and thereby prevented assembly of a ZP around growing oocytes [144]. An alternative explanation for the observation has recently been put forward that does not involve interference with secretion of nascent ZP proteins by truncated ZP1, but rather by affecting the crosslinking function of ZP1 [91].

Other studies also have attributed female infertility to GSV in *hZP1*. A heterozygous missense mutation in exon-3 of *hZP1* was identified in an infertile patient whose oocytes lacked a ZP [140]. The mutation resulted in His replacing Arg109 at the N-terminus of ZP1. Similarly, a compound heterozygous mutation consisting of a point mutation and deletion in *hZP1* was identified in an infertile woman whose oocytes lacked a ZP [142]. The mutation in exon-5 resulted in synthesis of hZP1 stopping at Glu292 and a 2 bp deletion in exon-7 also resulted in a premature SC and synthesis of hZP1 stopping at Ile386.

Several additional studies also led to identification of GSV in *hZP1* in infertile females who had abnormal oocytes. For example, a missense mutation in exon-2, Trp83Arg, was found in a patient with degenerated oocytes and an abnormal or no ZP, and in another patient a nonsense mutation with a premature SC in exon-8, Trp471>X, had a similar phenotype [138]. A compound heterozygous mutation, Arg61Cys and Ile390Thrfs*16, was found to be associated with abnormal oocytes and no ZP since replacement of Arg61 with Cys was predicted to be deleterious to hZP1 and a frameshift mutation introducing an SC in exon-7, Ile390*fs*404X, resulted in a 234 aa deletion at the C-terminus of hZP1 [136,137]. In another case, two frameshift mutations in *hZP1* resulted in premature SCs in exon-1, Gly57Aspfs*9, and exon-7, Ile390Thrfs*16, and apparently disrupted interactions between hZP proteins and caused degeneration of oocytes [136]. Missense mutations, Val570Met and Arg410Trp, were identified in two infertile females that had no oocytes or oocytes lacking a ZP [141]. Similarly, a compound heterozygous mutation with premature SCs in exon-9, Cys478>X, and exon-12, Asp592Glyfs*29, and a frameshift mutation in exon-3, His170Ilefs*52, were identified that possibly resulted in a truncated hZP1 that interfered with ZP formation [141]. A mutation in exon-2 of *hZP1*, G199>T (E67>X), resulted in a truncated protein of 67 aa that impaired secretion and ZP assembly [139]. In certain cases, GSV in *hZP1* affected its ZPD, a region of all ZP proteins considered critical for proper secretion of nascent ZP proteins and proper assembly of a ZP around growing oocytes [8,10,11,78].

### 8.2. Infertile Women and Mutant hZP2, hZP3, or hZP4 Genes

GSV in *hZP2* and *hZP3* of infertile women can also result in synthesis of hZP proteins that are unable to undergo normal secretion and assembly during oocyte growth. An infertile woman was found to have a heterozygous missense mutation in *hZP2*, exon-19 Arg698>X, with insertion of an SC at aa 698 and a heterozygous frameshift mutation in *hZP3*, exon-8 Arg349Leu>X, followed by an SC [146]. Both mutations resulted in the synthesis of truncated ZP proteins, hZP2 lacking a TMD and hZP3 lacking a CTP. Three other cases of GSV in *hZP2* have been described in which Cys372 was changed to Ser, Arg533 to Ser, and Cys566 to Arg [138,141]. All these changes occurred at conserved aa residues in the ZPD of hZP2, aa 371–637. A homozygous frameshift mutation in *hZP2* gave rise to a deletion variant in exon-11, c.1235_1236del, resulting in an altered aa and a truncated hZP2, Gln412Argfs*17 [145]. The mutation was located within the N-terminal half of the ZPD and was predicted to impede the interaction between hZP2 and hZP3, resulting in a thin ZP. A heterozygous missense variant in exon-2 of *hZP3* also was identified as a change of Ala134 to Thr; a change proposed to cause empty follicle syndrome and female infertility [140,147]. A similar missense mutation in exon-5 of *hZP3*, Arg255Gly, was found in a female with primary infertility [141]. In both cases the mutations occurred in the ZPD of hZP3, aa 45–304. A heterozygous mutation in exon-3 of *hZP3*, C518>G (S173C), located in the ZP-C subdomain, changed a highly conserved Ser to Cys and resulted in oocytes that lacked a ZP [148]. Two separate heterozygous variants were found in *hZP4*, a G298>A mutation in exon-3 (D100N) and a G1330>C mutation in exon-10 (V444L), and in each case the oocytes were surrounded by a thin and irregular ZP [149]. The mutations affected 2 highly conserved aa and caused impaired assembly and function of the ZP.

## 9. Summary Points

(i) The ZP is an atypical ECM that surrounds all mammalian oocytes, eggs, and preimplantation embryos and plays vital roles during oogenesis, fertilization, and preimplantation development. A ZP appears around growing oocytes during oogenesis while arrested in the dictyate stage of meiosis and is shed by expanded blastocysts just prior to implantation in the uterus. The presence of a ZP is required for normal oocyte growth, follicle development, and species-restricted fertilization. The ZP also assists in prevention of polyspermic fertilization and protects cleavage-stage embryos as they traverse the female reproductive tract on their way to the uterus.

(ii) The ZP is composed of either three or four proteins, ZP1-4, each with a unique polypeptide chain that is heterogeneously glycosylated with both N- and O-linked oligosaccharides. ZP2-4 are monomers and ZP1 is a dimer interconnected by a disulfide bond. ZP1-4 are encoded by single-copy genes located on different chromosomes that are only expressed by oocytes during their growth phase. *ZP* genes are hypomethylated in oocytes where they are expressed, as compared to somatic cells where they are not expressed. Genes encoding ZP proteins are conserved such that a *ZP* gene promoter from one mammal (e.g., human) can utilize the transcriptional machinery of oocytes from a different mammal (e.g., mouse). Expression of *ZP* genes in growing oocytes is regulated by *cis*-acting sequences located close to the transcription start-site and by *trans*-acting factors, certain of which are restricted to growing oocytes.

(iii) ZP proteins are synthesized, packaged in unusually large secretory vesicles, and secreted into the extracellular space. There ZP2 and ZP3 form heterodimers that, in turn, polymerize into long fibrils which exhibit a structural repeat. ZP fibrils are crosslinked by ZP1 and/or ZP4. ZP1-4 each have a ZPD that consists of two subdomains, ZP-N and ZP-C, that have an Ig-like 3-dimensional structure. The presence of both ZP2 and ZP3, but not ZP1 or ZP4, is required for assembly of ZP fibrils and matrix during oocyte growth. Polymerization of ZP proteins is regulated by sequence elements such as the CTP, EHP, and IHP that prevent premature polymerization of nascent ZP proteins in growing oocytes. Proteolytic cleavage of inhibitory sequence elements results in exposure of polymerization elements, such as subdomain ZP-N, and assembly of crosslinked ZP fibrils. ZP proteins possess some amyloid-like structural and physical features and have been proposed to be functional amyloids.

(iv) Failure to assemble a ZP around growing oocytes during oogenesis results in female infertility. Infertility is due to a paucity of growing oocytes and antral follicles in ovaries which results in very few, if any, ovulated eggs in oviducts. Such a situation occurs when *mZP* genes are inactivated by either homologous recombination or insertional mutagenesis or when *hZP* genes undergo point, missense, or frameshift mutations. In many instances, these *hZP* gene mutations result in the insertion of premature SCs, synthesis of truncated ZP proteins lacking sequence elements required for protein polymerization, and failure to assemble a normal ZP around growing oocytes. In the absence of a ZP, the stability of gap junctions between oocytes and surrounding follicle cells is reduced, thereby compromising transfer of nutrients, metabolites, and other molecules essential for oocyte and follicle growth and development.

The role of the ZP during mammalian oogenesis, fertilization, and preimplantation development has been of interest to clinicians and research scientists since the middle of the 19th century. An arsenal of contemporary methodology, from biochemistry to genetics to X-ray crystallography, has been employed during the past 40–50 years in order to reveal many significant features of ZP structure and function. Consequently, today we have a much deeper understanding and appreciation of the composition, genesis, evolution, and role of this unique and remarkable ECM. It is likely that further X-ray crystallographic, electron cryo-microscopic, advanced imaging, and other contemporary experimental approaches will provide answers to a variety of remaining questions about the ZP and its essential roles during mammalian development.

## Figures and Tables

**Table 1:** The Zona Pellucida Domain of ZP1–4.

	ZPD ^2^	ZP-N ^3^	ZP-C ^4^
^1^ mZP1	271 (271–541)	99	149
^1^ hZP1	270 (279–548)	99	148
mZP2	267 (364–630)	96	148
hZP2	267 (371–637)	96	148
mZP3	260 (45–304)	96	135
hZP3	259 (45–303)	97	133
hZP4	275 (188–462)	99	151

^1^ m, mouse; h, human. ^2^ Number aa in the ZPD (aa position of ZPD). ^3^ Number aa in the ZP-N subdomain. ^4^ Number aa in the ZP-C subdomain that includes the IHP.

**Table 2:** Peimary Structure of Mouse (m) and Human (h) ZP proteins.

ZP Protein	Polypeptide Length (aa)	Single Sequence (aa)	ZP Domain (aa)	Consensus Furin Cleavage-Site (aa)	Transmembrance Domain (aa)	Trefoil Domain (aa)
mZP1	623	1–20	271–542	545–548	591–611	225–266
hZP1	638	1–25	279–549	552–555	602–622	234–274
mZP2	713	1–34	364–630	632–635	684–703	-
hZP2	745	1–38	371–637	639–642	717–736	-
mZP3	424	1–22	45–304	350–353	387–409	-
hZP3	424	1–22	45–303	349–352	388–408	-
hZP4	540	1–19	188–462	463–466	505–526	141–183

**Table 3:** Phenotypes of *ZP1,2,3* Null Female Mice.

Genotype	Fertility	Zona Pellucida	References
Wild-type	Fertile	Normal	-
*ZP1^-/-^*	Reduced Fertility	Abnormal	[118]
*ZP2^-/-^*	Infertile	None	[119]
*ZP3^-/-^*	Infertile	None	[120,121]
*ZP3^+/-^*	Fertile	Thin	[83]

**Table 4:** hZP1–4 Mutations in Infertile Human Patients.

*hZP1* Mutations	Location of Mutation	Status of Zona Pellucida	References
G57Dfs*9	exon-1, SC in NI befroe TD	none	[136]
R61C	exon-1, NI befroe TD	none (?)	[137]
W83R	exon-2, NI befroe TD	abnormal/none	[138]
E67>X	exon-2, SC in NI befroe TD	none	[139]
RI09H	exon-3, NI befroe TD	none	[140]
H701fs*52	exon-3, SC between NI and TD	none	[141]
Q292>X	exon-5, SC in ZP	none	[142]
I386>X	exon-7, SC between ZP-N and ZP-C(linker)	none	[142]
I390fs404X	exon-7, SC between ZP-N and ZP-C(linker)	none	[143,144]
I390Tfs*16	exon-7, SC between ZP-N and ZP-C(linker)	none	[136,137]
R410W	exon-7, between ZP-N and ZP-C(linker)	none	[141]
W471>X	exon-8, SC in ZP-C	abnormal/none	[138]
C478>X	exon-9, SC in ZP-C	none	[141]
V570M	exon-11, between CFCS and EHP	none	[141]
D592Gfs*29	exon-12, SC between CFCS and TMD	none	[141]
***hZP2*** **Mutations**			
C372S	exon-11, ZP-N	thin/none	[141]
Q412Rfs*17	exon-11, ZP-N	thin	[145]
R533S	exon-15, ZP-C	normal/none	[138]
C566R	exon-16, ZP-C	abnormal/none	[138]
R698>X	exon-19, SC between CFCS and TMD	very thin/none	[146]
***hZP3*** **Mutations**			
A134T	exon-2, ZP-N	none	[140,147]
S173C	exon-3, ZP-C	none	[148]
R255G	exon-5, ZP-C	none	[141]
R349L>X	exon-8, SC at CFCS	very thin/none	[146]
***hZP4*** **Mutations**			
D100N	exon-3, NI	thin, irregular	[149]
V444L	exon-10, ZP-C	thin, irregular	[149]

Abbreviations: CFCS, concensus furin cleavage-site; EHP, external hydrophobic patch; h, human.

## Data Availability

Not applicable.

## Abbreviations

aa, amino acids; bp, base pairs; C-terminus, carboxy-terminus; CTP, carboxy-terminal propeptide; CFCS, consensus furin cleavage-site; cM, centimor-gan; CT, cytoplasmic tail; Cys, cysteine; ECM, extracellular matrix; EHP, external hy-drophobic patch; fg, femtograms; GSV, gene sequence variations; hZP, human ZP; Ig, immunoglobulin; IHP, internal hydrophobic patch; pI, isoelectric point; IVF, in vitro fertilization; kDa, kilodaltons; mRNA, messenger-RNA; MW, molecular weight; mZP, mouse ZP; nt, nucleotides; N-terminus, amino-terminus; PGCs, primordial germ cells; pg, picograms; pl, picoliters; Pro, proline; SC, stop-codon; SS, signal sequence; SV, se-cretory vesicles; TD, trefoil domain; TMD, transmembrane domain; ZP, zona pellucida; ZPD, zona pellucida domain; ZP-C, ZPD carboxy-terminal subdomain; ZP-N, ZPD amino-terminal subdomain.
